# m6A modification patterns are associated with copy number burden and tumor immune landscape in thyroid cancer

**DOI:** 10.1186/s12902-023-01510-3

**Published:** 2023-12-06

**Authors:** Liangliang Cai, Tingting Liu, Hujia Hua, Xingyu Jiang, Li Qian

**Affiliations:** 1 Institute of Translational Medicine, Medical College, Yangzhou University, No. 48 East Wenhui Road, Yangzhou, Jiangsu 225009 PR China; 2 Jiangsu Key Laboratory of Experimental & Translational Non-coding RNA Research, Yangzhou, 225001 PR China; 3 Department of Orthopedics, The Affiliated Hospital of Yangzhou University, Yangzhou University, Yangzhou, 225000 PR China

**Keywords:** Thyroid cancer, N6-methyladenosine, Tumor immune microenvironment, Immunotherapy strategy

## Abstract

**Background:**

The association involving N6-methyladenosine (m6A) modification, molecular subtype and specific immune cell group in tumor microenvironment has been the focus of recent studies. The underlying function of m6A modification in thyroid cancer (TC) remains elusive.

**Methods:**

The m6A modification regulations, molecular character and tumor immune profile of 461 TC patients were explored and then the correlation between them were comprehensively evaluated. The m6Ascore was established using principal component analysis (PCA) to quantify the m6A pattern of individual TC patients. The prognostic significance of the m6Ascore was evaluated by multivariate Cox regression analysis.

**Results:**

Four m6Aclusters (mc1, 2, 3, 4)—characterized by differences in extent of aneuploidy, expression of immunomodulatory genes, mRNA or lncRNA expression pattern and prognosis were identified. T Preliminary validation of m6Ascore was a potential independent prognostic factor of TC involving in mc3. Finally, the prognostic value of the m6Ascore and its association with copy number variation (CNV) and tumor immune microenvironment (TIME) of TC in mc3 were verified.

**Conclusions:**

The correlation between m6A modification, the copy number burden and tumor immune landscape in TC was demonstrated. A m6Acluster-mc3 with low m6Ascore and high CNV molecular subtype was identified with poor clinical prognosis, low infiltrating immunocyte and weak effector T cell. A three-gene clinical prognosis model for TC based on 4 m6a cluster expression was established. Understanding of TIME is enhanced by comprehensive assessment of m6A patterns in individual TC patients and gives a new insight toward improved immunotherapy strategies for TC cancer patients.

## Background

N6-methyladenosine (m6A), produced by methylation of N6 adenosine [1], can regulate multiple RNA-related biological processes, such as RNA stability [2], translation [3], alternative splicing [4, 5] and nuclear export [6]. The m6A modification is an equilibration process regulated by three class levels: writers (m6A methyltransferases consisting of 8 genes including ZC3H13, VIRMA, CBLL1, WTAP, RBM15/RBM15B, METTL3/14), 2 erasers (m6A demethylases consisting of ALKBH5 and FTO) and 14 readers (m6A-binding proteins by LRPPPRC, IGF2BP1/2/3, RBMX, YTHDC1/2, YTHDF1/2/3, HNRNPA2B1, HNRNPC, ELAVL1, FMR1) [7–9]. As an essential RNA modification, m6A regulated multiple important cellular processes, such as cellular differentiation, stem cell renewal and response to DNA damage [10]. With a consideration of m6A’s important role, aberrant expression of m6A regulators is explored to be associated with malignant cancer and the immune events including tumor development and tumor microenvironment (TIME) [10, 11].

In the clinical treatment of thyroid cancer (TC), immune checkpoint inhibitor therapy (ICT, mainly PD-1/PD-L1 monoclonal antibody therapy) is considered to be an important factor in tumor treatment due to its significant anti-tumor effect and limited side effects. It has great promise [12, 13], but not all the TC patients show the effective clinical response or even primary resistance to the ICT therapies [14]. In many malignant cancer types, a large number of tumor intrinsic, for example, when TIME is characterized by a high proportion of CD8 + T cell infiltration, an effective response to ICT therapy occurs [15, 16] while when CD8 + T cells When the cell infiltration is low, there is no response [17, 18]. In order to improve the efficacy and safety of immunotherapy, it is of great significance to explore the drivers of ICT clinical response in TC [19, 20]. Further research on the relevant molecular characteristics of clinical treatment strategies for immuno-oncology therapy will also be of great help for treatment optimization [21, 22].

The relationship between m6A regulators and immune cells has lately been the subject of several investigations. The METTL3-mediated m6A alteration enhanced the stimulation of DC-based T-cell and dendritic cells (DCs) responses by increasing the translation of specific immune genes [23]. T cells’ homeostasis and differentiation were disturbed when METTL3 was deleted. [24] Removal of YTHDF1 increased the antigen-specific CD8 + T cells’ antitumor response and improved the effectiveness of anti-PD-L1 treatment, according to Han et al. [25]. However, because of limitations in basic experiment, the preceding study is restricted to a small quantity of cell types and m6A regulators, whereas cancer formation and progression rely on interaction between numerous m6A RNA methylation regulators [9]. As a result, a thorough examination of the immunological landscape regulated by a range of m6A regulators would improve our overall knowledge of m6A regulators’ immunomodulatory (IM) influence on the TIME. The gastric cancer’s m6A modification patterns were recently analyzed thoroughly on the basis of numerous m6A regulators and systematically linked with the tumor immune landscape, showing that m6A modification pattern acts as a key part in TIME diversity in gastric cancer [26].

In this investigation, we integrated the clinical and molecular data of 461 TC patients to comprehensively evaluate the m6A modification pattern and TIME. Four distinct m6A modification regulation patterns were identified, and we were surprised to find that they had distinct molecular subtypes, immune characters and clinical prognoses, showing the key roles of m6A modification in the developments of individual tumor landscape in TC. We then quantified the m6A modification of individual TC patients by evaluating the gene patterns of m6A regulators.

## Methods

### Molecular and clinical data

RNA sequencing data (count and fpkm values) for gene expression analysis, genetic mutations (Mutect2), and clinical data were downloaded from the Genomic Data Commons (https://portal.gdc.cancer.gov/) [27]. The Ensembl gene IDs of the RNA-seq data were mapped to gene symbols by referring to an annotation file (https://www.gencodegenes.Org/human/release_22. html). The copy number variation (CNV) data were downloaded from the xena web tool (https://xena.ucsc.edu/) [28].

### Model-based clustering analysis for m6A regulators

Gene expression levels were quantified using the metric log2 (fpkm + 1), then used to identify m6A modification patterns based on the expression of 24 m6A regulators genes by model-based clustering analysis implemented in the R package/mclust. [29] In this package, the optimal number of clusters was determined based on the Bayesian information criterion (BIC).

### Gene set variation analysis (GSVA)

Gene set variation analysis, a non-parametric and unsupervised method commonly used for estimating pathway variations in the samples of expression datasets, was performed to explore the differences in biological processes among different m6A modification patterns [30]. The c2 .cp .kegg .v6.2 .symbols gene sets for GSVA were downloaded from the Molecular Signatures Database (MSigDB). A *p* < 0.05 was considered statistically significant.

### Identification of differentially expressed genes (DEG) among m6Aclusters

Based on published literature, RNA methylation is regulated by 24 genes, including 8 writers, 14 readers and 2 erasers were highlighted [7–9]. To identify genes related to m6A modification regulation, we classified TC patients into m6Aclusters based on the expression of 24 m6A genes. DEGs among these clusters were determined using the R package ‘limma’, which was applied using the raw fpkm values of RNA sequencing data. Genes with adjusted *p* < 0.05 and at least two-fold changes in expression were identified as DEGs.

### Construction of the m6A gene signature

We applied a methodology to quantify the m6A modification pattern (m6Ascore) of individual TC patients. The m6Ascore was established as follows. First, we extracted the overlapping DEGs among m6Aclusters and classified the TC patients into several groups using model-based clustering to analyze the overlapping DEGs. Univariate Cox regression analysis was performed to evaluate the prognosis of each overlapping DEG. Genes with a significant prognosis (*p* < 0.05) were extracted for further analysis. Next, principal component analysis (PCA) was performed to establish the m6A gene signature. We selected both principal components 1 (PC1) and 2 (PC2) as signature scores. Finally, the m6Ascore was defined using a method similar to Genomic Grade Index [26, 31, 32]:

$$\mathrm m6\mathrm{Ascore}\;=\mathrm\Sigma(\mathrm{PC}1\mathrm i\;+\;\mathrm{PC}2\mathrm i)$$

where i is the expression of overlapping genes with a significant prognosis of DEGs among m6Aclusters.

### Correlation between m6Ascore and other relevant biological processes

Spearman’s correlation analysis was performed to explore the correlation between m6Ascore and other relevant biological processes using the gene sets reported by Mariathasan et al., [18] including (1) antigen processing machinery (APM), (2) effector CD8 T-cell signature, (3) immune checkpoint, (4) nucleotide excision repair, (5) mismatch repair, (6) DNA replication, (7) DNA damage repair, (8) epithelial-mesenchymal transition markers, (9) Wnt targets, (10) pan-fibroblast transforming growth factor-β response signature, and (11) angiogenesis signature.

### Prediction of the potential chemotherapeutic agents

Genomics of Drug Sensitivity in Cancer (GDSC) is a public dataset containing information on drug sensitivity in cancer cells and molecular markers of drug response. Using the R/oncoPredict [33] package, GDSC2 gene expression profile and corresponding drug response information were downloaded to generate a ridge regression model that can be applied to transcriptomic data. Then the sensitivity scores were yielded to predict the half-maximal inhibitory concentration (IC50) of chemotherapy agents (Cisplatin and Paclitaxel) in TC patients.

### Tumor Immune Dysfunction and Exclusion (TIDE) for immune landscape evaluation

TIDE (http://tide.dfci.harvard.edu/, accessed on 15 March 2022), [34] an online algorithm for predicting the tumor immune dysfunction and exclusion status, was performed based on the transcriptome data. The m6Ascore of each patient in TCGA-THCA cohort was calculated and regarded as the m6Ascore grouping criteria based on model-based cluster by R/mclust package. Notably, the difference of immune signature (such as CAF, IFNG, CD8 and CTL) score between the groups was compared using Chi-square test.

### Statistical analysis

Statistical significance for 3 or more groups was estimated using the Kruskal-Wallis test and association between categorical variables was explored using the χ2 test. The correlation coefficient was calculated via Spearman’s correlation analysis. The Kaplan-Meier method was used to generate survival curves and the log-rank test was used to determine the statistical significance of differences. The oncoplot function of R package/maftools [35] was used to depict the mutation landscape of TCGA-THCA cohort. All tests were two sided, and *p* *<* 0.05 was regarded as significant. All analyses were performed with R software V.4.1.0 (http://www.R-project.org).

## Results

### The 24 m6A regulators in TC: molecular characteristics and clinical relevance

**Fig. 1:**
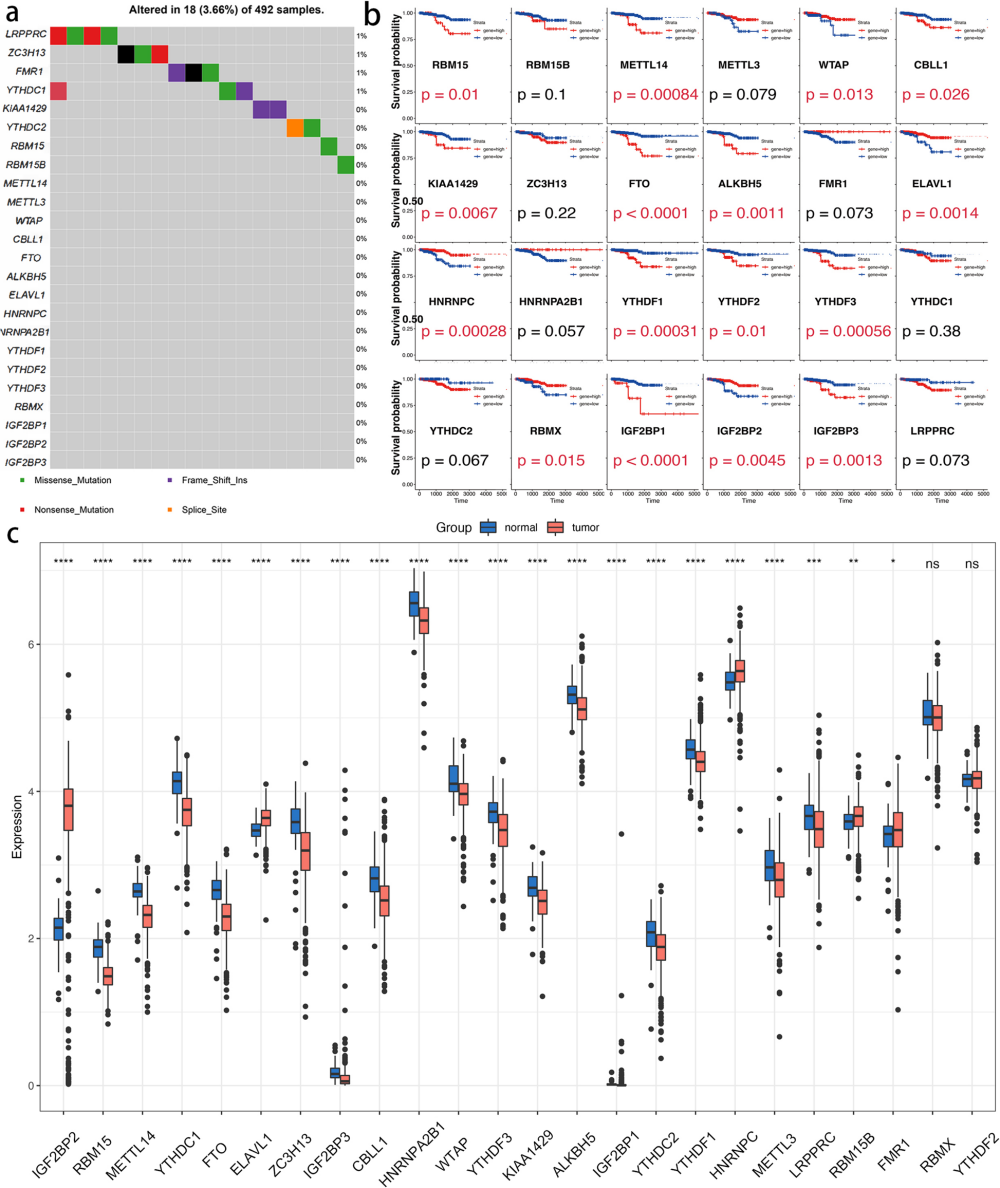
Clinical relevance and molecular characteristics of m6A regulator genes in TC. **A** The mutation landscape of 24m6A regulator genes in 492 TCs; **B** The overall survival of high or low expression of 24 m6A regulators in TCs; **C** The gene expression alterations among m6A regulators; Tumor (normal) was indicated in red (blue). ANOVA test: The asterisks represented the statistical *p* value (*p <0.05; **p <0.01; ***p <0.001)

The frequency of 24 m6A regulator changes in TC was investigated using somatic mutations. Only 18 of 492 samples had m6A regulator mutations, indicating that a complete average mutation frequency of m6A regulators was extremely low (Please see in Fig. 1a). The survival curve of the 24 m6A regulators was then examined, and it was shown that 16/24 m6A regulators had a substantial influence (*p* < 0.05) on TC patients (Please see in Fig. 1b). The m6A regulators’ mRNA expression levels in TC and surrounding tissues were also investigated, and it was discovered that 22 of the 24 m6A regulators were differently expressed (Please see in Fig. 1c). The expressional differences in m6A regulators were significantly diverse between TC and surrounding tissues, indicating that m6A regulator expression imbalance plays a critical role in formation and progression of TC. Furthermore, the activity of genes is not remote, showing that there is a collaboration in m6A regulators in cancer [36, 37]. These findings suggest that m6A regulators of RNA methylation play critical roles in the formation of TC.

### The m6A modification patterns mediated by 24 m6A regulators

**Fig. 2:**
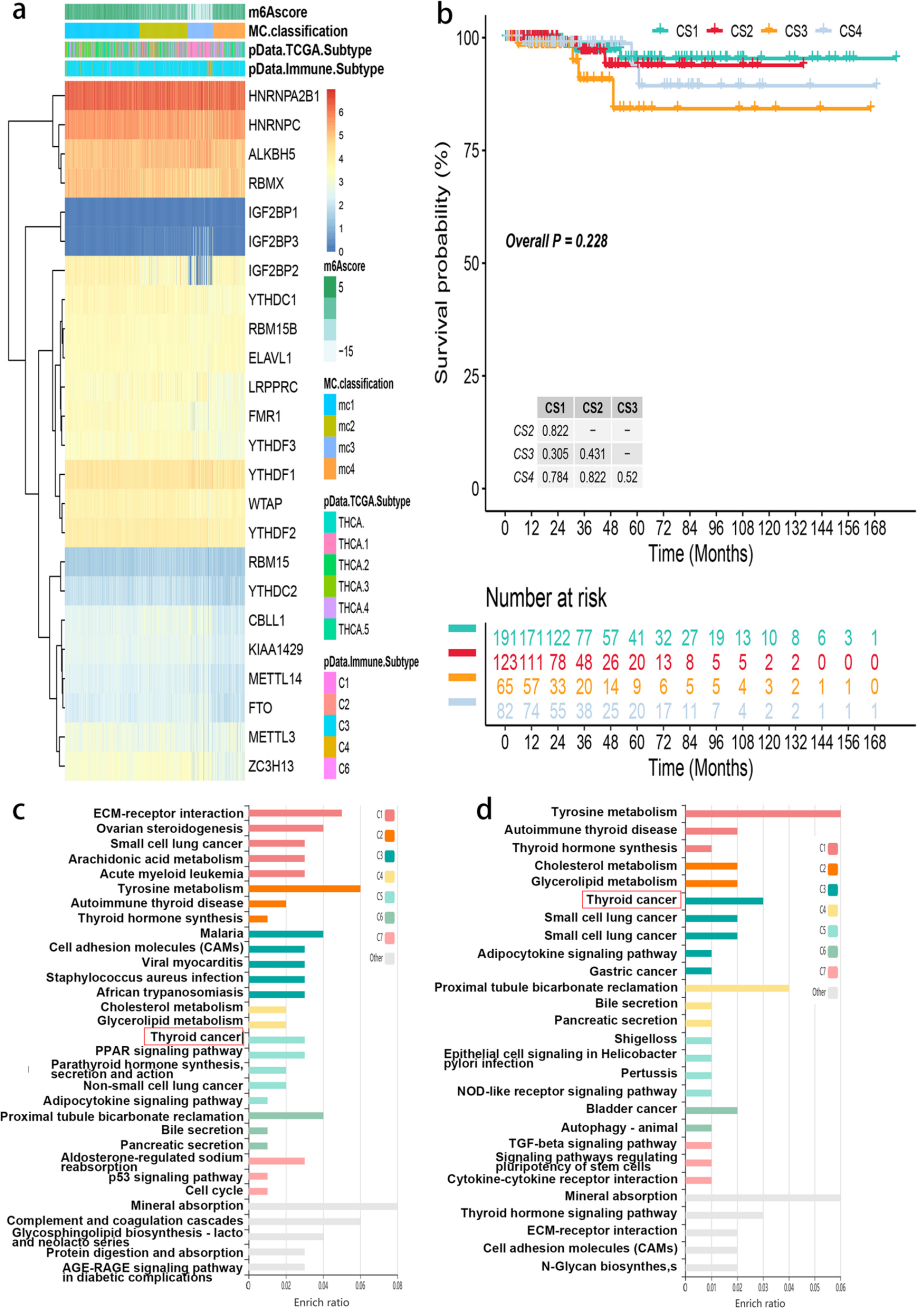
m6A modification patterns in TC and biological characteristics of m6A subtypes. **A** Model-based clustering of TC yields four subtypes in the TCGA-THCA dataset. MC1, cluster1; MC2, cluster2; MC3, cluster3; MC4, cluster4; **B** Comparison of prognosis among four m6A subtypes (Kaplan-Meier analysis); **C**, **D** 192 Over-lap DEGs and 128 Cox regression substantially DEGs enriched in KEGG pathways

The 24 m6A regulators’ expression was used to categorize TC patients using model-based clustering. We found four different RNA methylation modification patterns (called m6Aclusters mc1–mc4), with 118 cases in m6Acluster-c1, 129 cases in m6Acluster-c2, 53 cases in m6Acluster-c3, and 85 cases in m6Acluster-c4 (Please see in Fig. 2a). Favorable factors for overall survival (OS) (IGF2BP2) and risk factor for OS (IGF2BP3) were among the m6A regulators with the largest variations across subtypes (YTHDC1, RBMX, METTL14 and FTO). IGF2BP2, CBLL1 and FMR1 expression levels were low in m6Acluster-mc3, whereas YTHDF1, LRPPRC and IGF2BP3 expression was high. As a result, it’s no surprise that m6Acluster-mc3 had the poor prognosis (Please see in Fig. 2b).

To understand the m6A alteration pattern in individual TC patients, we performed a relatively accurate assessment using the m6Ascore approach. With the help of the limma program of R software, 192 DEGs associated with m6A isoforms can be found. On this basis, we assessed the prognosis of 128 genes in m6A subtype-associated DEGs using univariate Cox regression. Among these different m6A modification patterns, GO analysis allowed us to investigate the activity of KEGG pathway processes [38]. In DEGs and Cox regression substantially DEGs, they were notably enriched in pathways linked to thyroid cancer related terms, such as thyroid hormone production, lung cancer, and autoimmune thyroid disease, as depicted in (Please see in Fig. 2c and d). Meanwhile, immune-related pathways such as the NOD-like receptor signaling pathway, TGF-beta signaling route, and cytokine-cytokine receptor interaction were shown to be overrepresented among the implicated pathways.

### Immune characteristics and subtype identification in distinct m6A modification patterns

**Fig. 3:**
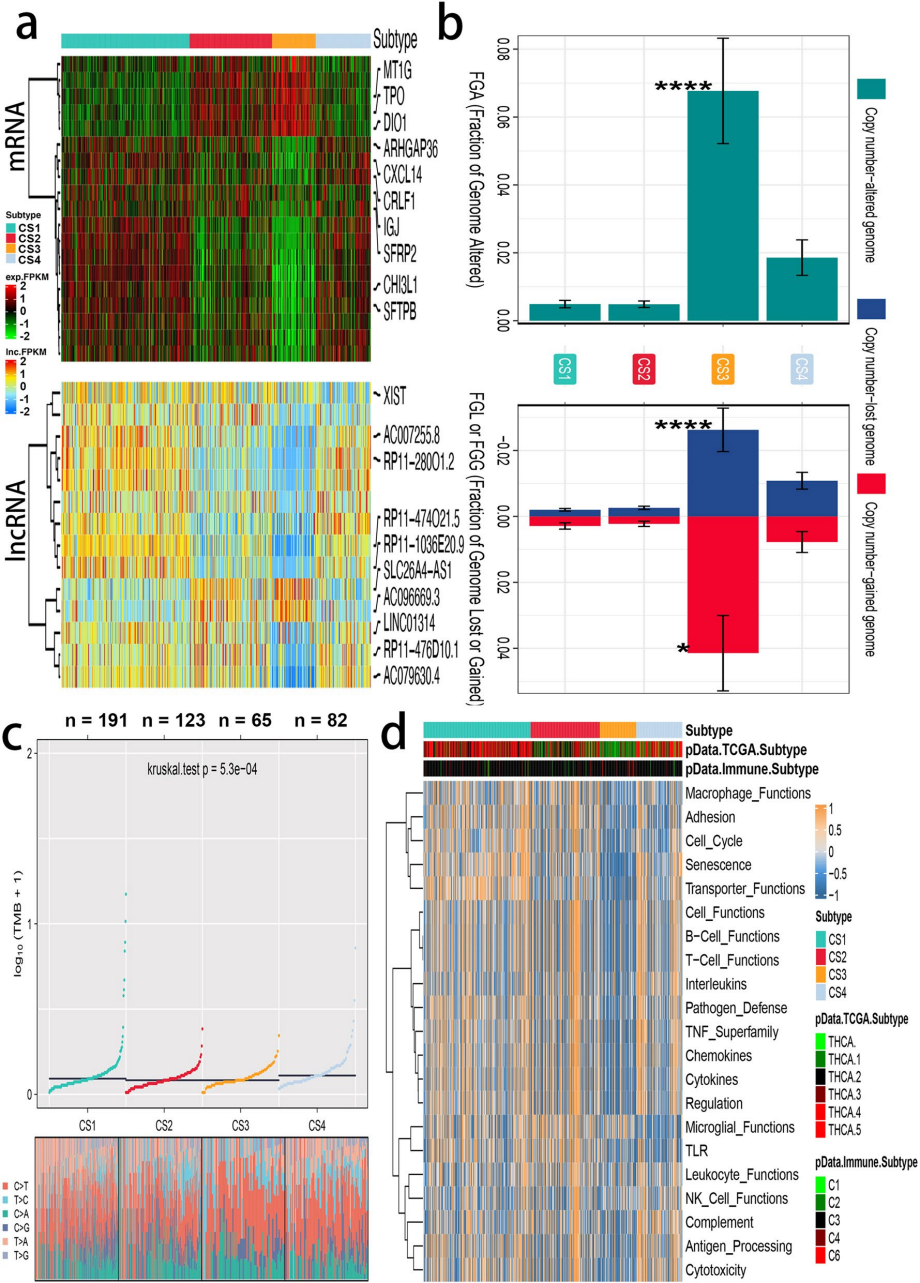
Different responses of immune cells are enriched in the four subtypes of thyroid cancer. **A** Molecular subtypes in distinct m6Aclusters. From top to bottom: mRNA expression (median normalized expression levels); lncRNA expression (median normalized expression levels); **B** Barplot of fraction genome altered among four identified subtypes of thyroid cancer in TCGA-THCA cohort; **C** Comparison of TMB and TiTv among four identified subtypes of thyroid cancer in TCGA-THCA cohort; **D** Heatmap of enrichment score of gene set of interest for four identified subtypes in TCGA-THCA cohort; Heatmap plot showing the different immune related functions between m6A subtypes

Thorsson et colleagues [39] investigated the pan-cancer immune landscape and eventually found the six immune subtypes (C1–C6) considered for determining the immune response patterns and have consequences for future immunotherapy research. In most TC patients, the immune subtype C3 was enriched, which is characterized by lower levels of overall CNVs. Low to moderate tumor cell growth, increased Th17, and aneuploidy than the other immune subtypes. Surprisingly, the four unique methylation modification levels showed different C3 immune subtype proportions, with m6Acluster-c3 having lowest (96.14%), followed by m6Acluster-c1 (90.68%), and c2 (57.27%) (p 0.001). The immunological properties of various m6A modification patterns were next investigated in further detail. In comparison to the other clusters, m6Acluster-c3 had a high ITH, and lower levels of aneuploidy and overall CNVs (Please see in Fig. 3a, b and c). The aneuploidy score and overall CNVs were highest in m6Acluster-c2, as were the proliferation rate and ITH, and the macrophage signature was conquered by M0 macrophages. Th17 was increased, tumor cell proliferation was low, ITH was low, and aneuploidy and overall CNVs were low in m6Acluster-c3.

Now that there is much consensus on the importance of IM for cancer immunotherapy, a variety of IM antagonists and agonists have been studied in clinical oncology [40]. It was further found that relevant research on IM immunotherapy can only be advanced by understanding their expression in different patterns of m6A alterations. After studying the expression of IM genes in the m6A subtype (Please see in Fig. 3d), it was found that almost all function were poorly expressed in m6Acluster-c3 especially in immune, such as T function, B function, APC processing, Macrophage functions.

### Construction of the m6A gene signature and evaluated the immune landscape was significantly associated with m6Ascore

**Fig. 4:**
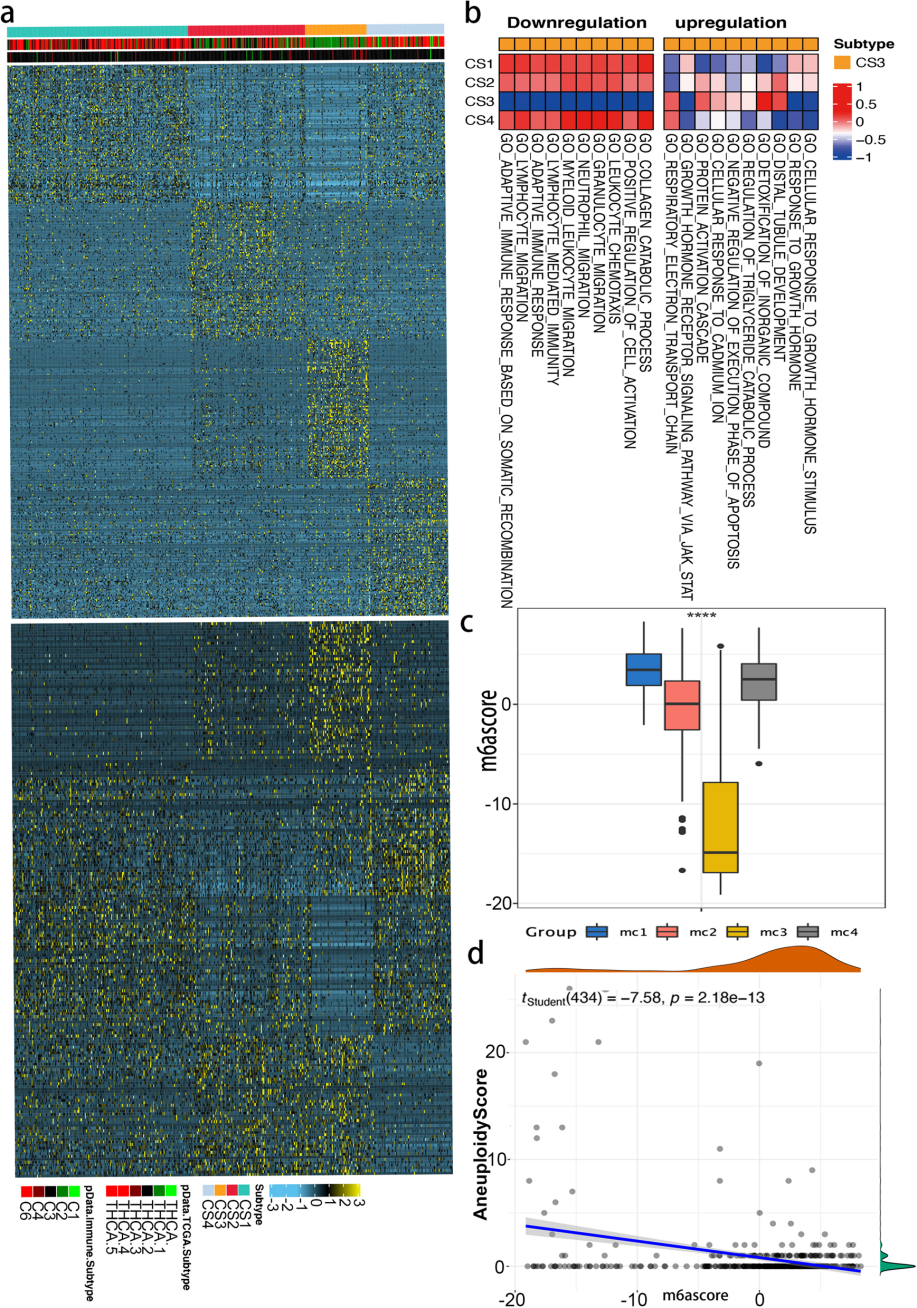
The immune landscape in distinct m6A modification patterns. **A** Heatmap of subtype-specific upregulated and downregulated biomarkers using limma for 4 identified subtypes in TCGA-THCA cohort; **B** GSVA of subtype-specific upregulated pathways (left). GSVA of subtype-specific downregulated pathways in TCGA-THCA cohort (right); **C** Boxplot showing the different m6aScore between m6A subtypes. ANOVA test: The asterisks represented the statistical *p* value (*p <0.05; **p <0.01; ***p <0.001); **D** Scatterplot with marginal distributions overlaid on the axes and results from statistical tests in the subtitle for m6ascore and cnv aneuploid score

For further exploration, an essential step is to characterize the functional pathways of different m6Acluster subtypes and potential predictive biomarkers. Starting with differential expression analysis (DEA), the data suggested the discovery of subtype-specific up- or down-regulated biomarkers. Biomarkers for each m6Acluster subtype were selected from the most DEGs sorted by log2Fold.so These biomarkers should pass the R/limma analysis to identify subtype-specific downregulated in Fig. 4a top and upregulated in 4a bottom biomarkers.

Likewise, on the basis that GSEA was run for each subtype based on its corresponding DEA results, we were able to identify functional pathways using a gene set context that included all gene sets derived from GO biological processes (c5.bp. v7.1.symbols.gmt) Heatmap analysis of subtype-specific downregulated biological pathways (Please see in Fig. 4b left) using limma package for 4 identified subtypes in TCGA-THCA and upregulated pathways (Please see in Fig. 4b right). To better demonstrate the molecular features of the m6A gene signature, we analyzed the differences between m6Asore by boxplots. Furthermore, the student *t* test showed a significant difference in m6Ascore among m6Aclusters (Please see in Fig. 4c). It was also shown that m6Ascore was negatively correlated with AS (*r*= − 0.22, *p* < 0.001). (Please see in Fig. 4d).

### The m6Ascore subtypes guided chemotherapy strategies and immune landscape evaluation

**Fig. 5:**
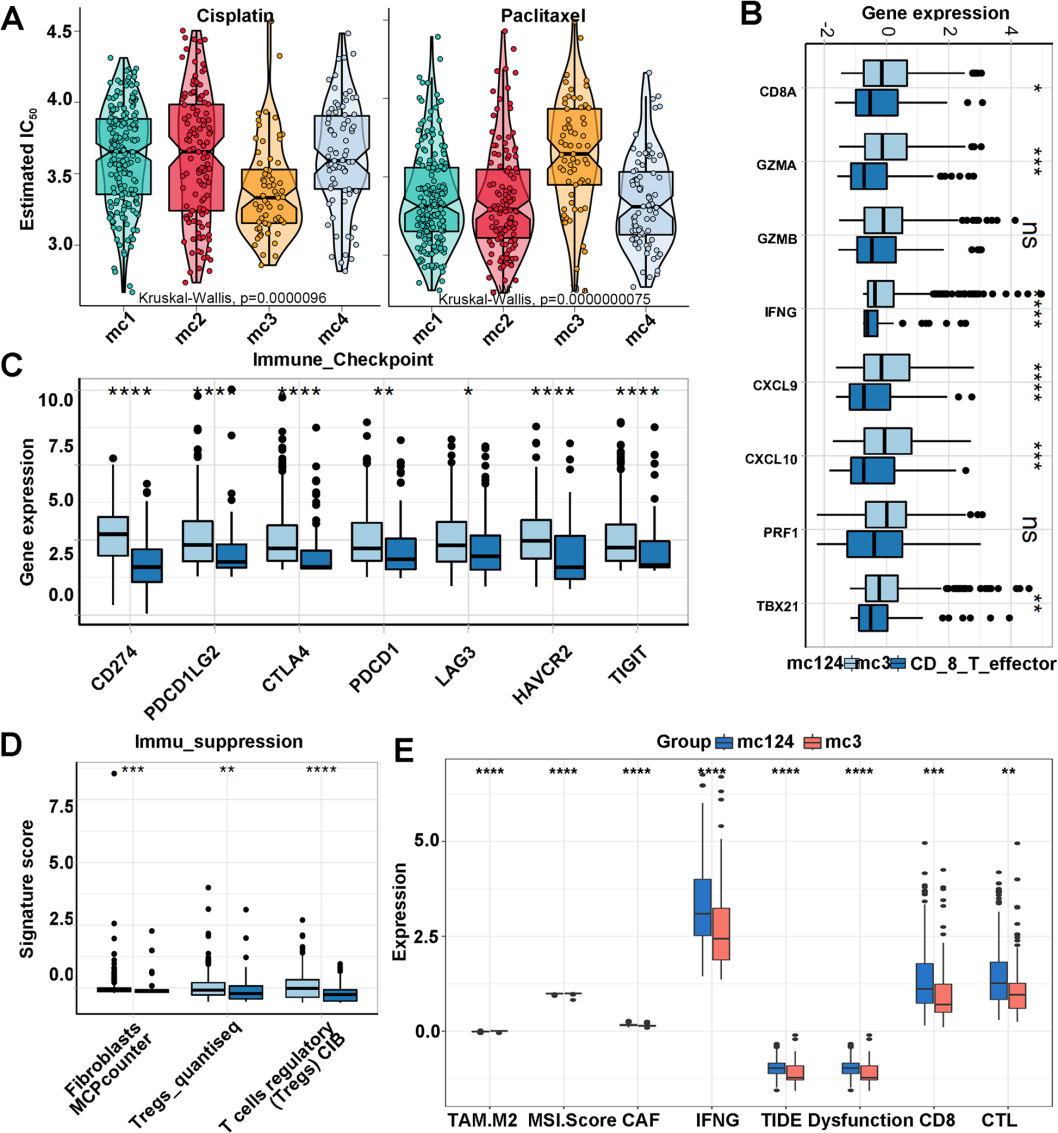
The m6Ascore subtypes guided chemotherapy strategies and immune landscape evaluation. **A** Boxviolins for estimated IC50 of Cisplatin and Paclitaxel among 4 identified m6Ascore subtypes in TCGA-THCA cohort. *p <0.05; **p <0.01; ****p <0.0001; **B** The RNA expression of eight CD8+ T cell effector genes in m6Ascore-mc3 and mc124 groups; **C** The immune checkpoint gene expression levels in m6Ascore-mc3 and mc124 groups; **D** The signature fibroblast, Treg and T cells regulatory score of immune suppression in m6Ascore-mc3 and mc124 groups; **E** The difference of the tumor immune dysfunction and exclusion score between the m6Ascore-mc3 and mc124 groups

For further explain the other functions of the classifier and the stability test of the classifier well, we dig deeper into the classifier function and verify the repeatability and stability of the classifier using drug sensitive prediction and immune status evaluation based on robust bioinformatics tools. Immunogenic cell death prompted by certain chemotherapy agents and subsequent tumor-specific immune response can determine the anticancer treatment effect of traditional cytotoxic drugs [41] and can also be used to sensitize tumors to checkpoint blockade, so the optimal combination of chemotherapy and immunotherapy warrants further exploration. Based on the 24 m6A gene sets, we generated m6Ascore, from which we could hypothesize that chemotherapy status might correlate with m6Ascore levels. Significance was found after comparing the estimated cis and pax sensitivities between the two subtypes. The OncoPredict package was used to predict the drug sensitivity score of m6Ascore-mc1234 group, and the sensitivity score was positively correlated with the IC50 values of cisplatin and paclitaxel. Our analysis revealed that IC50 were lower in patients who underwent Cisplatin chemotherapy (Please see in Fig. 5a left) and higher when Paclitaxel (Please see in Fig. 5a right).

The exploring expression of diverse m6A alteration patterns is required to progress this study. The functions based on the expression of IM regulators in the m6Acluster-c3 with three m6Acluster-c124 subtypes were investigated (Please see in Fig. 5b). CD8 + T cell effecter genes such as CD8A, GZMA, IFNG, CXCL9, CXCL10 and TBX21 were significantly down-regulated (*p* < 0.05) in the m6Acluster-c3 group, suggesting the decrease efficiency for T cells to recognize antigens and the inflammation and antitumor immunity. CD8 + T cell effecter genes was a novel biomarker with high sensitivity in predicting immunotherapy efficacy, and we identified its positive correlation with in the m6Acluster-c3. The activation of inhibitory checkpoint molecules prevents cancer cells from damage and attack so they can serve as promising targets for cancer immunotherapy. We investigated the immune checkpoint genes expression in THCA specimens and uncovered that CD274 (PD-L1), PDCD1, PDCD1LG2, CTLA4, HAVCR2, LAG3 and TIGIT were significantly low-expressed (*p* < 0.05) in the m6Acluster-c3 group (Please see in Fig. 5c). The immune suppression signature score of THCA specimens were calculated using the ESTIMATE package. The m6Acluster-c3 correlated with down signature scores (Please see in Fig. 5d), indicating the low levels of stromal and immune cells in the iTME. Tumors with m6Acluster-c3 correlated with elevated levels of multiple immune infiltration. In a nutshell, the complicated iTME of THCA was characterized by the mixture of tumor and antitumor cells, as well as the coexistence of immune activation and suppression. For further explore the relationship between m6Ascore and immune status, TIDE analysis was performed to predict the immune landscape in m6Acluster mc124 and mc3 groups. We calculated the tumor-intrinsic signature (CAF, IFNG, CD8 and CTL) scores of the TC patients and the analysis indicated that m6Ascore-related signatures were remarkably down-regulated in (Please see in Fig. 5e).

### Establishing a clinical prognosis model for thyroid cancer based on 4 m6a cluster expression

**Fig. 6:**
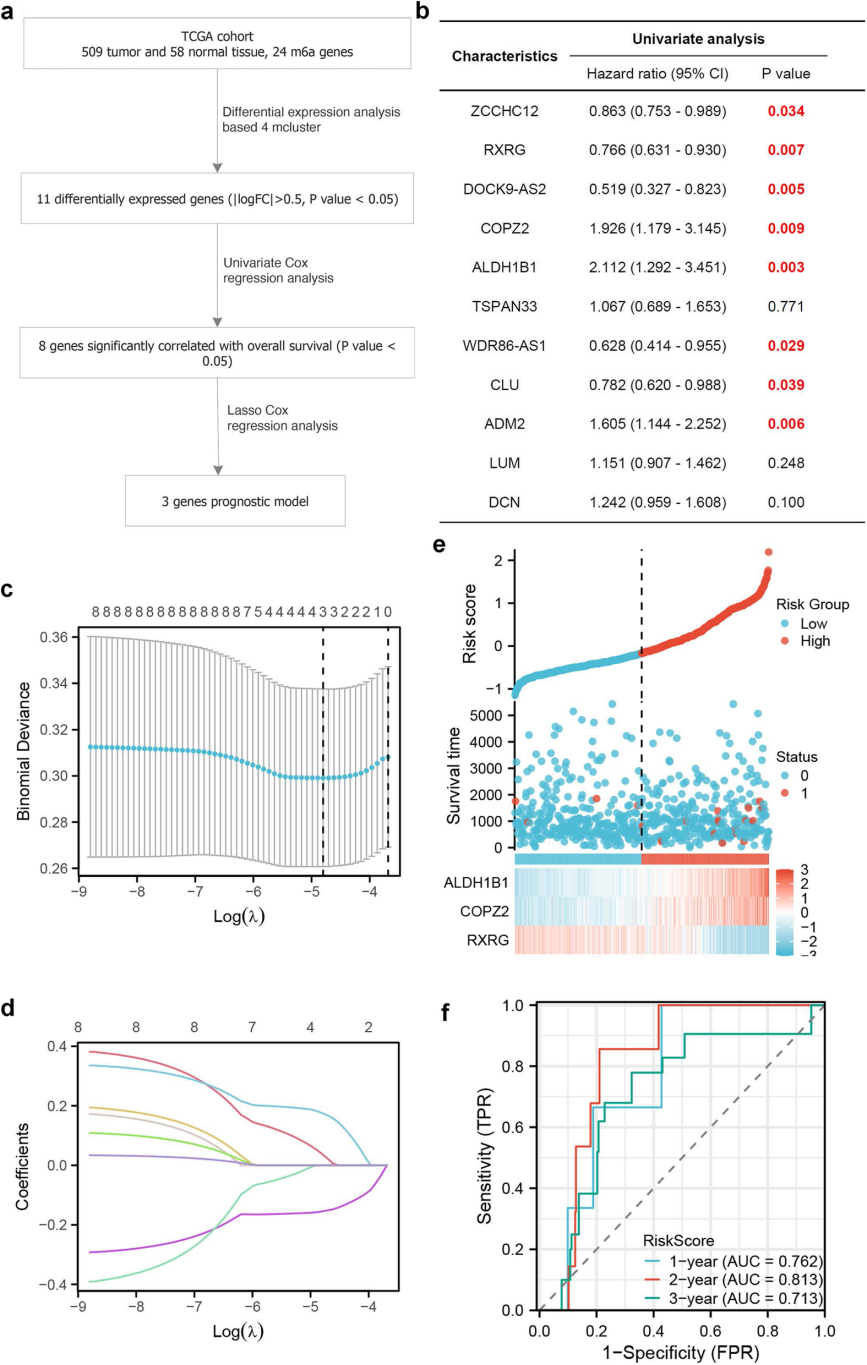
Analytical flow and results of the gene expression study in the TCGA cohort. **A** Flowchart of data processing and analysis; **B** Univariate analysis showcasing genes with their Hazard Ratios and *p*-values; **C** Lasso regression analysis; **D** Coefficient profiles of genes over Log(λ); **E** Risk score distribution, survival status, and gene expression heatmap for the derived 3-genes prognostic model; **F** ROC curve for the prognostic model predicting 1, 2, and 3-year survival

Based on the expression patterns of 4 m6a clusters, we further established a clinical prognosis model for thyroid cancer. In our analysis of the TCGA cohort, represented in Fig. 6a, which encompasses 509 tumor and 58 normal tissues with 24 m6a genes, differential expression analysis based on 4 clusters identified 11 significantly differentially expressed genes (|logFC|>0.5, *p* value < 0.05). Univariate Cox regression analysis, depicted in Fig. 6b, pinpointed 8 genes significantly correlated with overall survival. Subsequent application of Lasso Cox regression analysis, as illustrated in Fig. 6c and d, yielded a refined 3-genes prognostic model. The univariate analysis further highlighted genes like ZCCHC12, RXRG, and DOC9K-AS2 due to their substantial *p*-values, suggesting potential prognostic relevance. Figure 6e presents the risk score distribution, survival status, and a gene expression heatmap of the 3-genes prognostic model, demarcating two distinct risk groups: ‘High’ and ‘Low’. Lastly, Fig. 6f showcases the ROC curve for this model, predicting 1-year, 2-year, and 3-year survival, validating its accuracy with corresponding AUC values.

## Discussion

Published research studies had reported that m6A genes showed their crucial biological [42] and clinical functions [43] on tumor development, clinical therapeutic resistance and immune oncology response via cross-work among the m6A regulators [44]. Currently, the effects of m6A modification patterns on the TIME were explored in gastric cancer. [26] Jianzhong Hou et al.’s work [45] showed the importance to study m6A in TC. They evaluated the expressed of m6A regulators between tumor and normal samples, and correlation expression levels with clinical parameters. In our study, the role of m6A modification in the molecular subtype and immune landscape of TC was profiled to deep our knowledge of the immune oncology response based on TIME and provide more potentially effective ICT clinical treatment strategies.

The success of tumor immunotherapy depends on the induction of immune effector mechanisms, and CD8-positive T lymphocytes are an important part. It had been reported that METTL3 restrains papillary thyroid cancer progression via m(6)A/c-Rel/IL-8-mediated neutrophil infiltration [46]. Moreover, it is difficult for patients receiving ICT to obtain biopsy tissue during treatment, and the expression of CD8 + T cell effector genes has become an important clinical treatment evaluation tool. Our study has found that the m6Acluster-c3 in THCA samples can limit the infiltration of T cells, resulting in the blockage of the process of presenting antigens in tumor immunity, thereby inhibiting the immune function of T cells and helping the immune escape of tumor cells. In addition, immune checkpoint genes also showed a positive correlation with the m6Acluster-c3, which jointly contributed to the immune escape of tumor cells. Therefore, we can think that the m6Acluster-c3 is involved in the immunosuppressive tumor microenvironment of THCA.

Molecular genotyping based on genomic profiling [47–49] improves the clinical utility of TC patients in the future. Some research had found the unique RNA expression, SNPs and CNVs molecular character in TC by TCGA-THCA database [50, 51]. In current study, we identified m6A modification clusters with significantly different TIMEs based on 24 m6A gene regulators, of which 4 were significantly different: Differential drug treatment sensitivity, differences in aneuploidy, overall somatic copy number changes, expression levels of immune-related genes, and clinical outcome (OS). In our study, it can be concluded that the tumor growth rate is higher in m6Acluster-C1 because C1 shows enriched pathways associated with full immune activation and relatively high T cell function. Accordingly, it was not shown that c3 exhibited activated immunity but poor survival prognosis [52]. To accurately indicate m6A methylation levels, we applied a method called m6Ascore of individual TC patient to facilitate efficient and safe clinical application in TC patients. After an integrated analysis, it was revealed that m6Ascore may play a role in individualized immunotherapy as a potential independent prognostic factor for TC patients. In our study, negative correlation between m6Ascore and CNV burden has been found, which participated in the generation and metastasis of tumors, indicating the important role of m6A regulation in TC development. In this study, the clinical value of m6Ascore was validated in TC patients in cold immune state (m6Acluster-c3). It is well known that response to anti-PD-1/PD-L1 ICT therapy can be driven by pre-existing CD8 + T cell infiltration and high tumor mutational burden (TMB) [53, 54]. Thus, m6Ascore, a potential indicator of ICT therapy, can be added as one of them.

The study found a relationship between m6A alterations and copy number variation, and the link between the two and the immunological landscape of TC tumors was also investigated. Through in-depth analysis of m6A alteration patterns in individual TC patients, we have increased our understanding of the heterogeneity of TC tumor immune infiltration and the tumor immunological landscape, hoping to play a more role in the development of better novel immunotherapies for TC patient. We also stablished a three-gene clinical prognosis model for thyroid cancer based on 4 m6a cluster expression. We recognize that the sole reliance on the TCGA database, while a valuable resource, may not capture the full spectrum of variability present in the broader patient population. The limited sample size inherent to this singular dataset could potentially influence the robustness of our proposed m6A methylation modification regulator landscape for thyroid cancer. The pursuit of these future studies will be pivotal in enhancing the predictive power and clinical relevance of our findings, thereby contributing to the personalized treatment of thyroid cancer. Considering that our previous results lack clinical cohorts to verify, future exploration needs to be carried out on the basis of further verification of large cohort prospective clinical trials.

## Data Availability

All data used in this work can be acquired from the GDC portal (https://portal.gdc.cancer.gov/), Broad GDAC Firehose (https://gdac.broadinstitute.org/) and the website (https://gdc.cancer.gov/ about- data/ publications/ panimmune). All methods were performed in accordance with the relevant guidelines and regulations.
